# Elliptical heads result in increased glenohumeral translation along with micro-motion of the glenoid component during axial rotation in total shoulder arthroplasty

**DOI:** 10.1007/s00402-021-04018-4

**Published:** 2021-07-03

**Authors:** Lukas N. Muench, Cameron Kia, Matthew Murphey, Elifho Obopilwe, Mark P. Cote, Andreas B. Imhoff, Augustus D. Mazzocca, Daniel P. Berthold

**Affiliations:** 1 Department of Orthopaedic Surgery, UConn Health, Farmington, CT USA; 2 Department of Orthopaedic Sports Medicine, Technical University of Munich, Munich, Germany

**Keywords:** Humeral head, Elliptical, Spherical, Prosthesis design, Total shoulder arthroplasty, Micro-motion, Glenohumeral translation

## Abstract

**Introduction:**

Elliptical-shaped humeral head prostheses have recently been proposed to reflect a more anatomic shoulder replacement. However, its subsequent effect on micro-motion of the glenoid component is still not understood.

**Materials and methods:**

Six fresh-frozen, cadaveric shoulders (mean age: 62.7 ± 9.2 years) were used for the study. Each specimen underwent total shoulder arthroplasty using an anatomic stemless implant. At 15°, 30°, 45° and 60° of glenohumeral abduction, 50° of internal and external rotations in the axial plane were alternatingly applied to the humerus with both an elliptical and spherical humeral head design. Glenohumeral translation was assessed by means of a 3-dimensional digitizer. Micro-motion of the glenoid component was evaluated using four high-resolution differential variable reluctance transducer strain gauges, placed at the anterior, posterior, superior, and inferior aspect of the glenoid component.

**Results:**

The elliptical head design showed significantly more micro-motion in total and at the superior aspect of glenoid component during external rotation at 15° (total: *P* = 0.004; superior: *P* = 0.004) and 30° (total: *P* = 0.045; superior: *P* = 0.033) of abduction when compared to the spherical design. However, during internal rotation, elliptical and spherical heads showed similar amounts of micro-motion at the glenoid component at all tested abduction angles. When looking at glenohumeral translation, elliptical and spherical heads showed similar anteroposterior and superoinferior translation as well as compound motion during external rotation at all tested abduction angles. During internal rotation, the elliptical design resulted in significantly more anteroposterior translation and compound motion at all abduction angles when compared to the spherical design (*P* < 0.05).

**Conclusion:**

In the setting of total shoulder arthroplasty, the elliptical head design demonstrated greater glenohumeral translation and micro-motion at the glenoid component during axial rotation when compared to the spherical design, potentially increasing the risk for glenoid loosening in the long term.

**Level of evidence:**

Controlled Laboratory Study

## Introduction

Although anatomic total shoulder arthroplasty (TSA) has been shown to provide sufficient pain relief and improvement in shoulder function for patients with end-stage glenohumeral osteoarthritis, glenoid component loosening has been reported to be one of the main contributors to postoperative implant failure [6, 19, 23, 26]. With a prevalence varying widely between 27 and 94% of cases, peri-glenoid radiolucent lines have also been associated to poorer patient-reported functional outcomes following TSA [6, 19, 26].

As previous studies have identified eccentric loading along with the resulting rocking of the glenoid component as an important biomechanical factor for implant loosening, the glenoid design has been suggested to be critical for long-term stability and clinical survival [2–4]. Biomechanically, Voss et al. recently found that a pegged glenoid design showed significantly increased micro-motion during eccentric axial loading when compared to a keeled glenoid design [29]. Although with this suggesting that the glenoid design is important for initial fixation strength, Throckmorton et al. did not find significant differences in clinical and radiographic outcomes between the two designs [28]. More importantly, this inconsistency may imply that other factors further influence the development of glenoid loosening, including the design of the humeral head prosthesis.

Recent literature has described the humeral head to be more elliptical in shape, rather than a perfect sphere [8, 11, 15]. As implants resembling the native anatomy may restore joint kinematics and ensure durability most sufficiently, the use of elliptical prosthetic head designs has become more popular [5, 13, 17]. In a dynamic shoulder model, elliptical and spherical heads demonstrated similar degrees of the total, internal, and external rotational range of motion (ROM) in shoulder arthroplasty [22]. However, a biomechanical study by Jun et al. found that non-spherical heads resulted in increased glenohumeral translation during axial rotation in the coronal, scapular, and forward elevation plane at various abduction angles when compared to spherical heads [18]. While an increased translation may resemble native kinematics more accurately, this may also lead to more eccentric loading on and greater micro-motion of the glenoid component, potentially resulting in implant failure over time.

Thus, the purpose of this study was to investigate if there would be a difference in glenohumeral translation and micro-motion of the glenoid component during axial rotation when comparing elliptical and spherical prosthetic heads in the setting of TSA. The authors hypothesized that the elliptical prosthetic head design would result in significantly greater glenohumeral translation along with micro-motion of the glenoid component compared to the spherical head.

## Materials and methods

Six fresh-frozen, cadaveric shoulders with a mean age of 62.7 ± 9.2 years (range 48–74 years) were used for the study (Science Care Inc., Phoenix, AZ, USA). As de-identified specimens were not considered to constitute human subjects research, prior Institutional Review Board approval was not required.

### Specimen preparation

After having been thawed overnight at room temperature, specimens were dissected free of skin, subcutaneous tissue, muscles and capsule. Following disarticulation, specimens underwent visual inspection to exclude those with moderate to severe osteoarthritis or bony defects. Under fluoroscopy control (Mini C-Arm, GE Medical Systems Inc.), a 2.0 mm K-wire was drilled parallel to the glenoid surface from posterior to anterior at the middle of the superior–inferior diameter. A second 2.0 mm k-wire was drilled from inferior to superior parallel to the glenoid. The scapula body was trimmed using an oscillating saw and potted in a custom rectangular box with the glenoid surface being aligned parallel to the floor. The humerus was shortened and all soft tissues were completely removed. It was then centered and potted in a poly-vinyl chloride (PVC) pipe (diameter, 3.8 cm; length, 7 cm) using bone cement, leaving only 2 cm of the proximal humeral shaft exposed, to minimize diaphyseal bending moments [16, 20].

### Surgical technique

All surgical procedures were performed by the same surgeon (L.N.M) to minimize performance bias. TSA was performed using an anatomic stemless implant (Eclipse system, Arthrex Inc., Naples, FL, USA) as previously described [7, 10]. Oriented along the specimen’s anatomic retrotorsion, two 1.6 mm K-wires were pre-drilled in line with the desired resection plane, exiting the opposite cortex at the boundary of the articular cartilage. Guided by the two K-wires, subsequent osteotomy was performed using an oscillating saw. After measuring the anterior–posterior dimension of the resected humeral head, the size of the baseplate (trunnion) was determined. The trunnion was then fixed to the resected humeral neck and a hollow screw was inserted. Additionally, the custom-made trunnion used for this study was secured with a small, protruding spike, to allow for easily switching the different prosthetic heads during testing.

Glenoid replacement was performed using a keeled glenoid system (Univers II, Arthrex Inc., Naples, FL, USA). A glenoid guide was placed on the central axis of the exposed articular surface of the glenoid, with the guide handle being oriented in line with the anatomic slope of the anterior neck. Following preparation, a keeled glenoid implant was inserted in the created slot and impacted.

### Humeral head prosthetic design

Both elliptical and spherical prosthetic humeral heads were custom-made (Arthrex Inc., Naples, FL, USA). The designs, including equations for dimension width, radius of curvature, and height, were chosen according to previously published studies [13, 14]. A small hole in the under-surface allowed for securely placing the humeral head prosthesis on the protruding spike of the trunnion, avoiding rotation of the head prosthesis during testing and allowing for easily switching heads between testing conditions.

### Testing setup

**Fig. 1:**
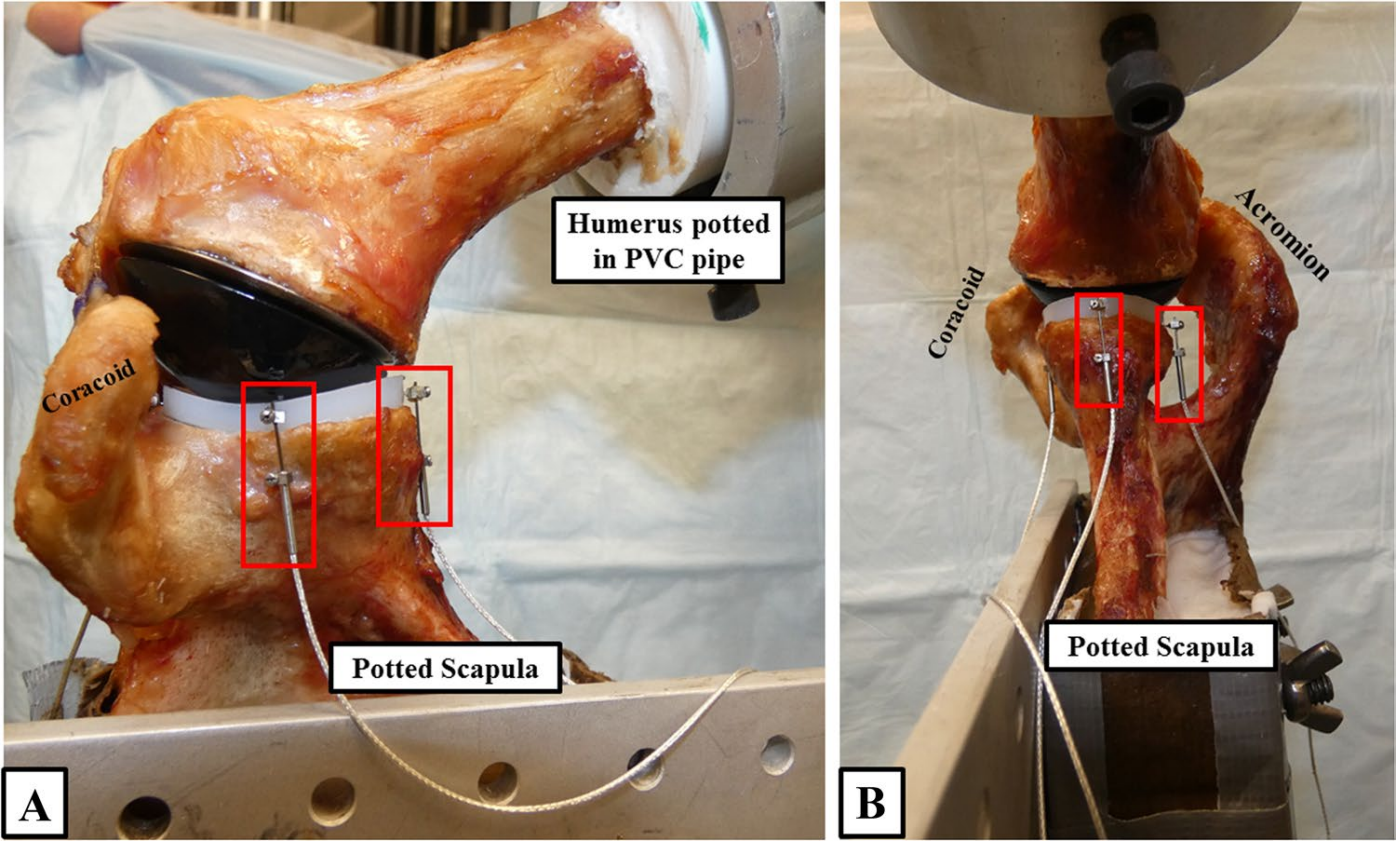
With the glenoid surface being in a horizontal position, the scapula is fixed to a vertical linear bearing translator and lever arm system on top of an X–Y table. The rotation of the humerus is in neutral position. To determine micro-motion of the glenoid component, four high-resolution DVRT (red boxes) are placed at the anterior, posterior, superior, and inferior aspect of the glenoid component. **A** Anterior view. **B** Inferior view

**Fig. 2:**
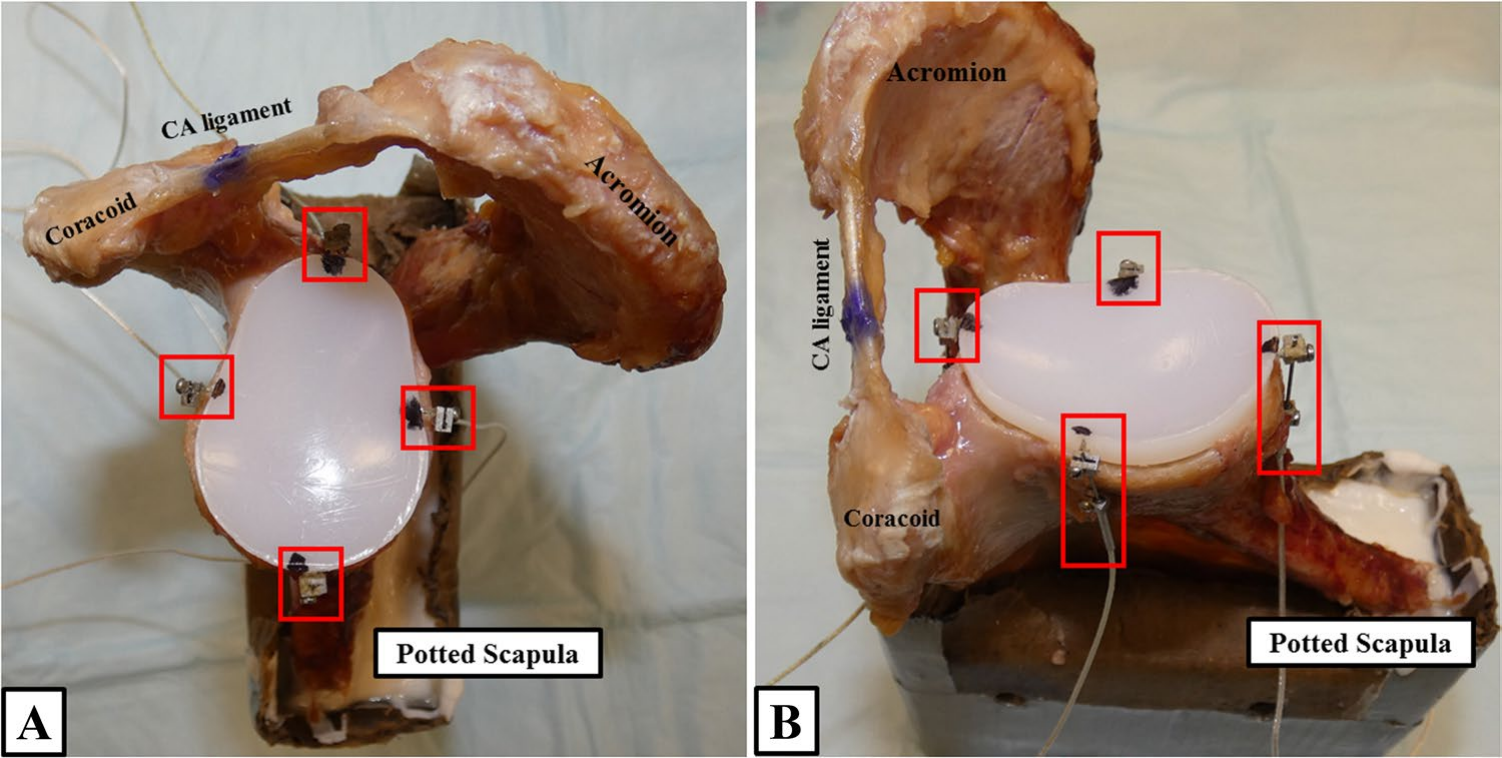
To determine micro-motion of the glenoid component, four high-resolution DVRT (red boxes) are placed at the anterior, posterior, superior, and inferior aspect of the glenoid component. **A** Bird’s-eye view. **B** Anterior view

The specimens were mounted to a validated shoulder testing rig as previously described, which allowed for positioning of the glenohumeral joint in 6 degrees of freedom [1, 20, 21, 24, 25]. With the glenoid surface being in a horizontal position, the scapula was fixed to a vertical linear bearing translator and lever arm system on top of an X–Y table, allowing for glenohumeral translation in the anteroposterior and superoinferior direction (Fig. 1). The rotation of the humerus was defined as neutral with the bicipital groove being aligned with the anterior margin of the acromion according to Selecky et al. [16, 27]. To determine micro-motion of the glenoid component, four high-resolution differential variable reluctance transducer (DVRT) strain gauges (Microstrain, Burlington, VT, USA) were placed at the anterior, posterior, superior, and inferior aspect of the glenoid component (Fig. 2) [29].

### Biomechanical testing

During testing, an axial compression load of 40 N was constantly applied via the lever arm of the X–Y table to center the joint [18]. According to Jun et al. [18], 50° of internal and 50° of external axial rotation were alternatingly applied to the humerus at 15°, 30°, 45°, and 60° of glenohumeral abduction in the scapular plane.

By means of a 3-dimensional digitizer (MicroScribe G2; Immersion) with a position accuracy of 0.23 mm, the position of the X–Y table was measured by carefully digitizing the center of a defined groove on the X–Y table without relevant influence by touching off with the digitizer. The position of the groove was determined at the beginning (start position) and the end (end position) of each application of internal or external rotation. Changes in the position represented the glenohumeral translation and were given in anteroposterior (*x*-axis) and superoinferior (*y*-axis) directions. In addition, overall compound motion during internal and external rotation was calculated as the square root of the sum of the squared anteroposterior (*x*-axis) and squared superoinferior (*y*-axis) translation.

After completion of translational testing for each condition, micro-motion of the glenoid component during internal and external rotation was assessed using the mounted DVRT strain gauges. The final position in internal or external rotation was maintained for five seconds to allow the measuring curve to form a plateau for analysis.

During evaluation of translation and micro-motion, internal and external rotations were each applied five times for every condition. Values of each specimen were then averaged and are presented as the final values. Throughout the entire testing, specimens were not removed from the testing rig, nor was the testing rig disassembled. To avoid selection bias, the order of glenohumeral abduction positions (15°, 30°, 45°, 60°) and head designs (elliptical or spherical) was randomly assigned.

### Statistical analysis

A power analysis was carried out to determine detectable differences in micro-motion, using standard deviations estimated from the literature as well as pilot data prior to this study [29]. Assuming a common standard deviation of 0.1 mm, a sample size of 6 specimens would provide 80% power to detect a 0.15 mm difference in micro-motion at an *α* level of 0.05.

Differences in micro-motion and translation between implants were assessed using multilevel mixed effects generalized linear models. A random intercept was used to account for specimens in different conditions. The gamma distribution was used to model micro-motion. For each analysis, the distribution of the residual was examined and found to conform to a normal distribution. Comparisons of marginal mean values were carried out and were adjusted for multiple comparisons using the Holm–Bonferroni sequential correction method in the presence of initial statistical significance. A *P* value of 0.05 was set to be statistically significant. All statistical analyses were conducted using Stata 15 software (StataCorp. 2017. Stata Statistical Software: Release 15. College Station, TX: StataCorp LLC).

## Results

### Micro-motion of the glenoid component

**Fig. 3:**
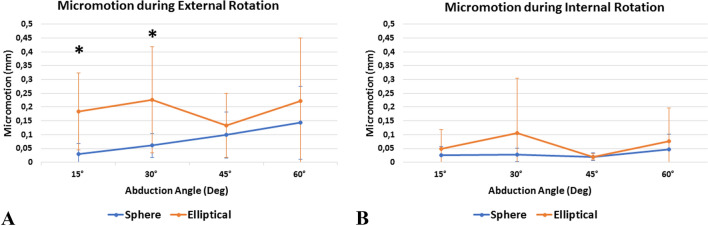
Total micro-motion (mm) at the glenoid component during external (**A**) and internal (**B**) rotation. * Indicates statistical significance

The elliptical humeral head design showed significantly more micro-motion in total (Fig. 3) and at the superior aspect of glenoid component during external rotation at 15° (total: *P* = 0.004; superior: *P* = 0.004) and 30° (total: *P* = 0.045; superior: *P* = 0.033) of abduction when compared to the spherical design (Table 1). Further, elliptical heads resulted in significantly more micro-motion at the posterior aspect of the glenoid component during external rotation in 15° of abduction (*P* = 0.004). However, there was no significant difference in micro-motion at the inferior and anterior aspects of the glenoid (*P* > 0.05, respectively). In addition, there were no significant differences in micro-motion during external rotation for both elliptical and spherical heads when comparing the resting position (15°) to the abduction angles of 30°, 45°, and 60° (*P* > 0.05, respectively).

**Table 1:** Amount of micro-motion (mm) in total as well as at the superior, anterior, inferior, and posterior aspect of the glenoid component during external (ER) and internal (IR) rotation at various abduction (ABD) angles

ABD Angle	Rotation	Design		Total	*P* value	Superior	*P* value	Inferior	*P* value	Anterior	*P* value	Posterior	*P* value
15°	ER	Elliptical	Mean ± SD	0.184 ± 0.140	0.004*	0.036 ± 0.039	0.004*	0.030 ± 0.022	0.112	0.029 ± 0.027	0.198	0.090 ± 0.097	0.004*
			Median	0.162		0.027		0.032		0.026		0.047	
			IQR	0.232		0.019		0.014		0.041		0.170	
		Sphere	Mean ± SD	0.031 ± 0.037		0.006 ± 0.005		0.008 ± 0.014		0.005 ± 0.006		0.012 ± 0.014	
			Median	0.015		0.007		0.002		0.002		0.002	
			IQR	0.026		0.005		0.000		0.003		0.020	
	IR	Elliptical	Mean ± SD	0.048 ± 0.070	0.888	0.028 ± 0.046	0.056	0.007 ± 0.010	0.999	0.006 ± 0.009	0.999	0.008 ± 0.009	0.907
			Median	0.013		0.005		0.002		0.002		0.002	
			IQR	0.032		0.014		0.003		0.000		0.014	
		Sphere	Mean ± SD	0.026 ± 0.031		0.006 ± 0.006		0.007 ± 0.012		0.003 ± 0.001		0.010 ± 0.013	
			Median	0.014		0.006		0.001		0.002		0.001	
			IQR	0.021		0.007		0.001		0.002		0.014	
30°	ER	Elliptical	Mean ± SD	0.227 ± 0.192	0.045*	0.038 ± 0.037	0.033*	0.029 ± 0.028	0.186	0.094 ± 0.102	0.076	0.067 ± 0.073	0.105
			Median	0.204		0.030		0.024		0.086		0.033	
			IQR	0.313		0.077		0.046		0.174		0.104	
		Sphere	Mean ± SD	0.061 ± 0.044		0.008 ± 0.005		0.008 ± 0.007		0.016 ± 0.021		0.029 ± 0.034	
			Median	0.041		0.009		0.007		0.007		0.019	
			IQR	0.076		0.008		0.004		0.019		0.030	
	IR	Elliptical	Mean ± SD	0.106 ± 0.199	0.168	0.022 ± 0.039	0.072	0.018 ± 0.032	0.156	0.026 ± 0.055	0.072	0.040 ± 0.073	0.089
			Median	0.027		0.005		0.003		0.003		0.010	
			IQR	0.041		0.018		0.017		0.005		0.025	
		Sphere	Mean ± SD	0.027 ± 0.024		0.004 ± 0.003		0.003 ± 0.003		0.007 ± 0.007		0.014 ± 0.018	
			Median	0.020		0.003		0.002		0.003		0.008	
			IQR	0.019		0.006		0.001		0.013		0.018	
45°	ER	Elliptical	Mean ± SD	0.134 ± 0.116	0.686	0.023 ± 0.041	0.450	0.028 ± 0.041	0.898	0.050 ± 0.079	0.999	0.033 ± 0.029	0.384
			Median	0.104		0.006		0.006		0.022		0.023	
			IQR	0.236		0.015		0.051		0.043		0.031	
		Sphere	Mean ± SD	0.099 ± 0.083		0.013 ± 0.022		0.018 ± 0.021		0.031 ± 0.043		0.037 ± 0.063	
			Median	0.080		0.005		0.010		0.006		0.012	
			IQR	0.160		0.008		0.025		0.075		0.028	
	IR	Elliptical	Mean ± SD	0.020 ± 0.012	0.888	0.003 ± 0.003	0.999	0.003 ± 0.002	0.999	0.006 ± 0.011	0.999	0.008 ± 0.006	0.321
			Median	0.015		0.002		0.002		0.002		0.007	
			IQR	0.016		0.003		0.003		0.002		0.007	
		Sphere	Mean ± SD	0.020 ± 0.014		0.004 ± 0.004		0.003 ± 0.003		0.006 ± 0.008		0.008 ± 0.008	
			Median	0.020		0.002		0.002		0.002		0.004	
			IQR	0.020		0.006		0.003		0.002		0.010	
60°	ER	Elliptical	Mean ± SD	0.222 ± 0.228	0.686	0.055 ± 0.068	0.450	0.067 ± 0.091	0.550	0.064 ± 0.071	0.999	0.036 ± 0.026	0.218
			Median	0.164		0.018		0.020		0.038		0.024	
			IQR	0.315		0.110		0.147		0.142		0.040	
		Sphere	Mean ± SD	0.143 ± 0.131		0.038 ± 0.049		0.043 ± 0.053		0.043 ± 0.048		0.019 ± 0.010	
			Median	0.112		0.011		0.019		0.025		0.020	
			IQR	0.251		0.083		0.096		0.082		0.017	
	IR	Elliptical	Mean ± SD	0.077 ± 0.119	0.888	0.005 ± 0.004	0.999	0.028 ± 0.062	0.426	0.029 ± 0.062	0.999	0.014 ± 0.012	0.713
			Median	0.033		0.006		0.003		0.002		0.011	
			IQR	0.036		0.005		0.005		0.010		0.019	
		Sphere	Mean ± SD	0.047 ± 0.056		0.004 ± 0.003		0.008 ± 0.012		0.021 ± 0.039		0.013 ± 0.014	
			Median	0.026		0.004		0.004		0.006		0.009	
			IQR	0.036		0.004		0.005		0.011		0.019	

^*^Indicates statistical significance

During internal rotation, elliptical and spherical heads showed similar amounts of micro-motion at the glenoid component at all tested abduction angles (Table 1). Additionally, there were no significant differences in micro-motion for both elliptical and spherical heads when comparing the resting position (15°) to the abduction angles of 30°, 45°, and 60° (*P* > 0.05, respectively).

### Glenohumeral translation

Elliptical and spherical heads showed similar anteroposterior and superoinferior translation as well as compound motion during external rotation at all tested abduction angles (*P* > 0.05, respectively) (Table 2). In addition, there were no significant differences in translation during external rotation for both elliptical and spherical heads when comparing the resting position (15°) to the abduction angles of 30°, 45°, and 60° (*P* > 0.05, respectively).

**Table 2:** Anteroposterior and superoinferior glenohumeral translation (mm) as well as overall compound motion (mm) during external (ER) and internal (IR) rotation at various abduction (ABD) angles

ABD Angle	Rotation	Design		Anteroposterior (mm)	*P* value	Superoinferior (mm)	*P* value	Compound motion (mm)	*P* value
15°	ER	Elliptical	Mean ± SD	− 5.1 ± 3.3	0.196	0.6 ± 1.3	0.888	5.3 ± 3.2	0.189
			Median	− 5.4		− 0.1		5.9	
			IQR	3.3		1.3		3.2	
		Sphere	Mean ± SD	− 4.0 ± 2.0		0.5 ± 0.8		4.2 ± 1.8	
			Median	− 4.8		0.0		4.8	
			IQR	2.2		1.2		2.4	
	IR	Elliptical	Mean ± SD	7.7 ± 4.4	< 0.001*	− 0.1 ± 0.5	0.688	7.8 ± 4.3	< 0.001*
			Median	7.4		0.1		7.4	
			IQR	3.2		0.1		3.2	
		Sphere	Mean ± SD	6.0 ± 4.4		0.0 ± 0.1		6.1 ± 4.4	
			Median	5.1		0.0		5.1	
			IQR	2.7		0.1		2.7	
30°	ER	Elliptical	mean ± SD	− 5.0 ± 3.9	0.180	0.6 ± 1.4	0.980	5.2 ± 3.9	0.212
			Median	− 3.9		0.1		3.9	
			IQR	7.6		1.0		7.3	
		Sphere	Mean ± SD	− 4.0 ± 3.0		0.6 ± 1.5		4.2 ± 3.0	
			Median	− 4.2		0.1		4.2	
			IQR	3.6		0.5		4.6	
	IR	Elliptical	Mean ± SD	8.2 ± 3.1	< 0.001*	0.5 ± 0.9	0.082	8.3 ± 3.0	< 0.001*
			Median	7.4		0.1		7.4	
			IQR	2.9		0.7		2.9	
		Sphere	Mean ± SD	6.0 ± 3.9		0.0 ± 0.1		6.0 ± 3.9	
			Median	5.7		0.0		5.7	
			IQR	3.0		0.1		3.0	
45°	ER	Elliptical	Mean ± SD	− 4.3 ± 3.1	0.395	0.2 ± 0.6	0.666	4.4 ± 3.0	0.379
			Median	− 4.1		0.4		4.2	
			IQR	2.8		0.6		2.8	
		Sphere	Mean ± SD	− 3.7 ± 2.8		0.1 ± 0.5		3.8 ± 2.7	
			Median	− 3.3		0.0		3.4	
			IQR	2.7		0.1		2.7	
	IR	Elliptical	Mean ± SD	6.9 ± 3.6	0.004*	0.0 ± 0.2	0.910	6.9 ± 3.6	0.008*
			Median	7.1		0.0		7.1	
			IQR	2.3		0.2		2.3	
		Sphere	Mean ± SD	5.8 ± 3.5		0.0 ± 0.1		5.8 ± 3.5	
			Median	5.9		0.0		5.9	
			IQR	2.9		0.2		2.9	
60°	ER	Elliptical	Mean ± SD	− 4.1 ± 2.2	0.240	0.7 ± 0.7	0.651	4.2 ± 2.3	0.252
			Median	− 4.6		0.7		4.6	
			IQR	2.1		1.2		2.2	
		Sphere	Mean ± SD	− 3.2 ± 1.9		0.5 ± 0.7		3.3 ± 1.9	
			Median	− 3.2		0.2		3.3	
			IQR	3.4		0.8		3.3	
	IR	Elliptical	Mean ± SD	6.4 ± 3.3	0.004*	0.5 ± 0.9	0.867	6.4 ± 3.4	0.008*
			Median	6.2		0.1		6.2	
			IQR	2.6		0.1		3.0	
		Sphere	MEAN ± SD	5.2 ± 3.1		0.4 ± 0.9		5.3 ± 3.1	
			Median	5.4		0.1		5.4	
			IQR	2.7		0.1		3.1	

^*^Indicates statistical significance. Negative values indicate posterior or superior translation, respectively

**Fig. 4:**
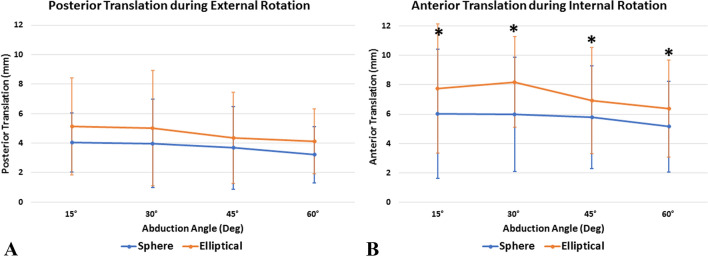
Amount of glenohumeral translation (mm) in the posterior direction during external rotation (**A**) and in the anterior direction during internal rotation (**B**). * Indicates statistical significance

**Fig. 5:**
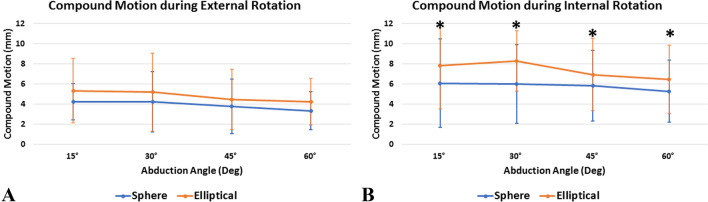
Amount of overall compound motion (mm) during external (**A**) and internal (**B**) rotation. * Indicates statistical significance

During internal rotation, the elliptical design resulted in significantly more anteroposterior translation (Fig. 4) and compound motion (Fig. 5) at all abduction angles when compared to the spherical design (Table 2). However, there was no significant difference when looking at superoinferior translation. Further, the elliptical head showed significantly less anteroposterior translation (*P* = 0.006) and compound motion (*P* = 0.012) during internal rotation at 60° of abduction when compared to the resting position (15°). All other comparisons for anteroposterior and superoinferior translation as well as compound motion were found to be non-significant (*P* > 0.05, respectively).

## Discussion

The most important finding of this study was that the elliptical head design resulted in significantly greater micro-motion at the glenoid component during external rotation at lower abduction angles when compared to the spherical design. However, during internal rotation, elliptical and spherical heads showed similar amounts of micro-motion at all tested abduction angles. In addition, elliptical heads showed significantly more anteroposterior glenohumeral translation and overall compound motion during internal rotation at all abduction angles when compared to the spherical design. More importantly, these biomechanical time-zero findings imply that the design of the humeral head prosthesis may further influence the development of glenoid component loosening in the long-term.

In the setting of TSA, glenoid component loosening has been reported to be one of the main contributors to implant failure, with peri-glenoid radiolucent lines having been associated to poorer postoperative functional outcomes [6, 19, 26]. Eccentric loading along with the resulting rocking (micro) motion of the glenoid component has been identified as an important biomechanical factor for subsequent implant loosening, thus the glenoid design has usually been suggested to be essential for ensuring long-term stability and clinical survival [2–4]. Although biomechanical studies have shown that the glenoid design is critical for initial fixation strength [29], clinical and radiographic studies demonstrating the superiority of one design over another are yet to be reported [28, 29]. More importantly, the inconsistency of clinical findings along with recent anatomic studies describing the humeral head to be rather elliptical in shape than a perfect sphere may imply that the design of the humeral head prosthesis may also have a considerable influence on the long-term stability of the glenoid component [8, 11, 13–15].

As implants resembling the native anatomy may ensure restoration of joint kinematics and durability most sufficiently, the use of elliptical prosthetic heads has garnered recent interest [13, 17, 18, 22]. Using a dynamic shoulder model, elliptical and spherical heads were found to achieve similar amounts of total, internal, and external rotational ROM in both hemi and TSA [22]. However, Jun et al. demonstrated that non-spherical heads resulted in increased glenohumeral translation during axial rotation in the coronal, scapular, and forward elevation plane at various abduction angles when compared to spherical heads [18]. Similarly, the present study found that the elliptical design showed significantly more anteroposterior glenohumeral translation and overall compound motion during internal rotation at all abduction angles.

While this increased translation resembles native glenohumeral kinematics more accurately, [17, 18] this may also lead to more eccentric loading on and greater micro-motion of the glenoid component. In this study, the elliptical head design demonstrated significantly greater micro-motion at the glenoid component during external rotation when compared to the spherical design. This was especially observed at lower abduction angles, where the glenohumeral joint is less constraint. As excessive micro-motion of the glenoid component has been suggested as a significant risk factor for glenoid loosening in the long-term, [2–4, 29] these biomechanical time-zero findings may be of clinical importance.

However, transferability into the clinical setting may be limited, as rotator cuff muscles and capsule were completely resected, to allow for accurate placement of the strain gauges directly on the glenoid. In presence of an intact rotator cuff and capsule, the spherical head may also be subjected to more translation, as it contains more physical material in the anteroposterior dimension compared to an elliptical head [13, 14]. Tightening of the anterior part (during external rotation) or posterior part (during internal rotation) of the capsule may push the spherical head more posteriorly or anteriorly during axial rotation [9, 12]. This may especially be observed at higher abduction angles with the glenohumeral joint being more constraint.

To date, clinical data regarding functional outcomes and long-term stability of shoulder arthroplasty using non-spherical humeral head implants are limited. Recently, Egger et al. reported no signs of component loosening along with significant improvement of shoulder function following non-spherical TSA [5]. However, the mean follow-up was only 43 months, which does not allow for drawing definite conclusions regarding implant longevity, and there was no control group of patients undergoing TSA using a spherical design [5]. Given the various morphologies of the humeral head, especially in the setting of osteoarthritis [8], may make the availability of both head designs important for functional outcomes.

There were several limitations to the study. Humeral head prosthetic design may show a different effect in vivo when compared to observations during laboratory cadaveric testing. In addition, the study is limited to the prior resection of rotator cuff muscles and capsuloligamentous structures, leaving the effect of these soft tissue restraints on glenohumeral translation and micro-motion unknown. As such, dynamic and static glenohumeral stabilizers including the negative intraarticular pressure and subsequent joint concavity compression were eliminated, usually having further implications on glenohumeral translation. However, removal of soft tissue was essential for accurate placement of the highly sensitive strain gauges, ensuring correct measurements without disruptive factors. Lastly, the inconsistencies in the anatomy of each individual specimen, with the humeral head either being more elliptical or spherical in shape, may have further influenced the results.

## Conclusion

With the use of an elliptical head design in TSA, significantly greater micro-motion at the glenoid component during external rotation at lower abduction angles can be expected when compared to the spherical design. Further, elliptical heads showed significantly more anteroposterior glenohumeral translation and overall compound motion during internal rotation at all abduction angles, potentially influencing glenoid component loosening over time.
